# Tandem ChoRE and CCAAT Motifs and Associated Factors Regulate Txnip Expression in Response to Glucose or Adenosine-Containing Molecules

**DOI:** 10.1371/journal.pone.0008397

**Published:** 2009-12-22

**Authors:** Fa-Xing Yu, Yan Luo

**Affiliations:** 1 Institute of Molecular and Cell Biology, Singapore, Singapore; 2 Department of Biochemistry, National University of Singapore, Singapore, Singapore; Universität Heidelberg, Germany

## Abstract

**Background:**

Thioredoxin interacting protein (Txnip) is a multifunctional protein involved in regulation of cell cycle events and cellular metabolism. The expression of Txnip is known to be induced by glucose, adenosine-containing molecules, and other physiological cues; however, the underlying regulatory mechanisms remain elusive.

**Methodology/Principal Findings:**

In this study, using promoter reporter, electrophoresis mobility shift (EMSA), and chromatin immuno-precipitation (ChIP) assays, we have identified an additional carbohydrate response element (ChoRE) on the promoter of Txnip gene, which functions cooperatively with the earlier identified ChoRE to mediate optimal Txnip expression. However, these two ChoREs are not sufficient to mediate the induction of Txnip expression by glucose or adenosine-containing molecules; and two CCAAT boxes, both of which can recruit nuclear factor Y (NF-Y) to the Txnip promoter, are also required for the induction. Accordingly, we have found that the function of ChoREs and associated factors is contingent on tandem CCAAT boxes, in that occupancy of the Txnip promoter by NF-Y is a prerequisite for efficacious recruitment of Mondo/MLX to ChoREs under glucose stimulation.

**Conclusions/Significance:**

Our findings suggest a synergy between the tandem CCAAT and ChoRE motifs and associated NF-Y and Mondo/MLX transcription factors in enhancing transcription from the Txnip promoter. This piece of information will be helpful for future dissection of molecular mechanisms governing the transcriptional regulation of Txnip, a glucose responsive gene.

## Introduction

Thioredoxin interacting protein (Txnip, also known as VDUP1 and TBP-2) is involved in many cellular and physiological processes [1]–[3]. First, Txnip can regulate cellular redox state by inhibiting the activity of thioredoxin [2], [4]–[6]. Second, Txnip is a candidate of tumor suppressors, as it can suppress cell cycle progression [7]–[13]. Third, suggesting a role in cell differentiation, the development of natural killer cells in Txnip deficient mice is disrupted [14]. Fourth, Txnip can promote apoptosis by modulating the function of the apoptosis signal-regulating kinase 1 (ASK1) [5], [15]. Finally, a wealth of accumulated information suggests an intimate involvement of Txnip in regulating glucose and lipid metabolism [16]–[29].

A number of physiological cues can dictate the efficacy of Txnip expression, which is inhibited by insulin [20], stimulated by glucocorticoid [30], [31], vitamin D [3], peroxisome proliferator-activated receptor (PPAR) agonist [32]–[34], transforming growth factor beta (TGF-β) [11], suberoylanilide hydroxamic acid (SAHA, an inhibitor of histone deacetylase [HDAC]) [13], [35], adenosine-containing molecules and certain stress signals [36], [37]. Most interestingly, the expression level of Txnip is tightly correlated with the extracellular glucose levels [22], [25], [27], [28], [37], and this glucose-induced Txnip expression negatively feeds back to the cellular glucose uptake system [17], [20]. Thus, Txnip may play an important role in glucose homeostasis.

While majority of Txnip-related research focuses on the Txnip function, the underlying mechanisms that govern Txnip gene transcription are largely unknown despite the identification of a lot of modulators for Txnip expression. For the glucose- or adenosine-containing molecules- induced Txnip expression, MondoA and Max-like protein X (MLX) function through carbohydrate response element (ChoRE) and are crucial for transmitting signals into the nucleus to activate the Txnip promoter [16], [37]; however, a detailed description of this signaling pathway and the regulatory mechanisms at the promoter level remains elusive.

Here, we show a dissection of the Txnip promoter in a detailed manner, which reveals a requirement for tandem ChoRE and CCAAT motifs that in conjunction with associated factors support optimal Txnip expression outputs in response to glucose or adenosine-containing molecules. We propose a model in which ChoRE- and CCAAT-associated factors, upon receiving signals from diverse physiological cues, induce dynamic changes at Txnip promoter in a synergistic manner and enhance Txnip expression.

## Results

### Txnip Promoter Regions Critical for Expression Induction by NAD^+^ or Glucose

**Figure 1:**
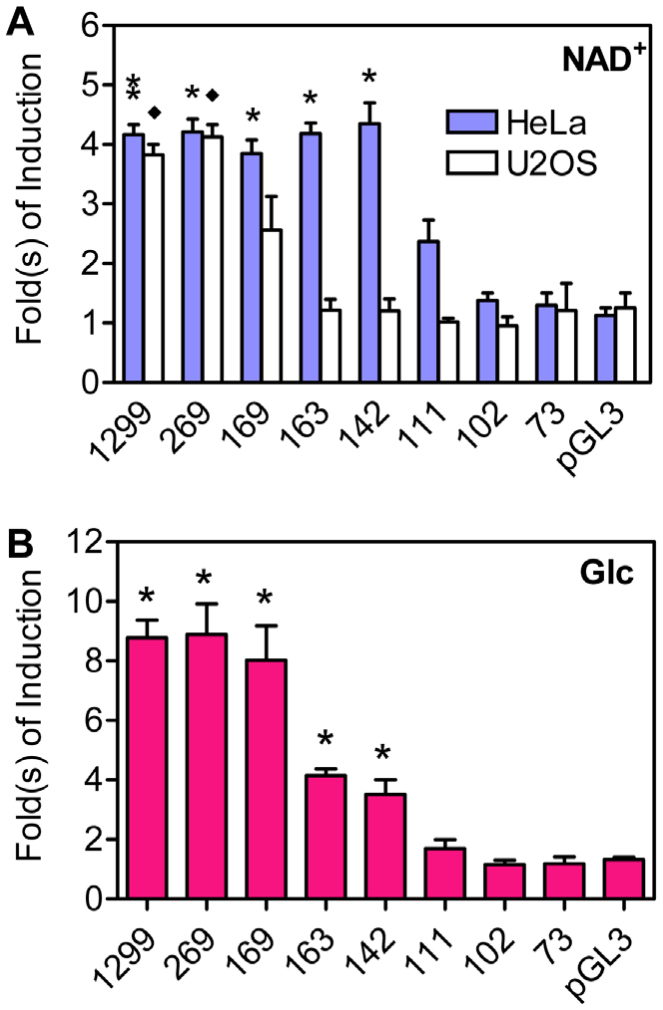
The induction of truncated Txnip promoters by NAD^+^ (A) or Glucose (B). Numbers under bars indicate length (upstream of the transcription start site) of corresponding Txnip promoters. Asterisk and diamond: significant different from pGL3 vectors, n = 2.

Previously, it has been shown that a 269 bp Txnip promoter fragment was sufficient, and in which the earlier identified ChoRE site (at ∼80 bp upstream of the transcriptional start site) was essential, to support the Txnip expression induction by glucose or adenosine-containing molecules [22], [37]; however, the minimal promoter that remains inducible by these molecules was not yet defined. To this end, we performed a more detailed promoter analysis. Txnip promoters with different lengths were generated and fused to a luciferase reporter gene. When ectopically introduced into HeLa cells, reporters with 142 bp or longer promoter sequences exhibited ∼4-fold, and a reporter with 111 bp sequences showed ∼2-fold, stimulation by NAD^+^; however, reporters with shorter sequences were not responsive to NAD^+^ (Figures 1A and S1A). In sharp contrast, in U2OS cells, NAD^+^ showed marginal effect on all ectopic reporters with less than 169 bp Txnip promoter sequences, and reporters with longer sequences exhibited similar stimulation by NAD^+^ as in HeLa cells (Figures 1A and S1B). Given that the previously identified ChoRE locates at ∼80 bp upstream of the transcriptional start site, this ChoRE is apparently not sufficient to support optimal induction of Txnip expression by NAD^+^.

We have also tested the effect of glucose on these Txnip promoters. In L6 cells, ectopic promoters with 111 bp or less sequences were not responsive to glucose; however, promoters with 142 bp or longer sequences showed 4-8-fold stimulation by glucose (Figures 1B and S1C). This suggests that the earlier defined ChoRE alone cannot support optimal induction of Txnip expression by glucose, and that some nucleotide sequences upstream of this ChoRE is also critical for the responsiveness of Txnip promoter to glucose.

**Figure 2:**
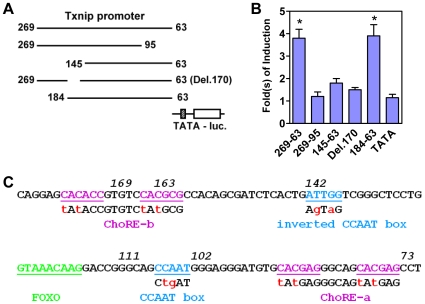
The minimal Txnip promoter sequence required for mediating the stimulatory effect of NAD^+^. (A) Txnip promoter segments with or without deletions were fused to a TATA box driven luciferase reporter, these hybrid promoters were used in (B). (B) the response of Txnip promoters to NAD^+^ treatment in U2OS cells; asterisk: significant different from TATA only promoter, n = 2. (C) Txnip promoter sequences critical for mediating the stimulatory effect of NAD^+^, and potential cis-regulatory elements in this region, numbers indicate the distance to the transcription start site. Modified nucleotides introduced into mutant Txnip promoters are indicated by lower case letters.

To identify minimal promoter sequences required for the induction of Txnip expression by adenosine-containing molecules, various Txnip promoter fragments were fused to a TATA-only core promoter-luciferase reporter gene (Figure 2A), and their responses to NAD^+^ were tested in U2OS cells. As shown (Figure 2B), a promoter fragment (184–63 bp upstream of the transcription start site) was sufficient to support the induction by NAD^+^, while a fragment (269–95) lacking an earlier defined ChoRE or fragments (145–63 or d170) lacking sequences surrounding the −170 positions did not respond to NAD^+^. We conclude that both the earlier defined ChoRE and nucleotide sequences near the −170 positions are critical for the induction of Txnip expression by adenosine-containing molecules.

The responses of the Txnip expression to extracellular glucose or adenosine-containing molecules are general phenomena that have been observed in diverse mammalian cell lines from different tissue origins; however, the fold of response to the adenosine-containing molecules was most prominent in U2OS cells, and the fold of response to glucose was most dramatic in L6 cells ([37] and data not shown). Thus, in most experiments, we respectively used U2OS or L6 cell lines to test the Txnip promoter activities in cells treated by adenosine-containing molecules or glucose.

### Tandem ChoREs on Txnip Promoters

We examined the Txnip promoter sequences (184–63 bp) and found that, apart from the known ChoRE (CACGAGggcagCACGAG; ∼80 bp upstream of the transcription start site), the nucleotide sequences around the −170 region (CACACCgtgtcCACGCG; Figure 2C) mimicked a degenerate ChoRE, which is defined as two E-boxes (CACGTG) separated by 5 nucleotides [38]. Thus, an additional candidate ChoRE sequence on the Txnip gene promoter was identified; for convenience, we dubbed the previously identified ChoRE (∼80 bp) as ChoRE-a and the newly identified ChoRE (∼170 bp) as ChoRE-b (Figure 2C).

Bioinformatics efforts allowed us to conclude that Txnip is vertebrate-specific, and obtain genomic sequences of Txnip promoters of diverse species from fishes to humans. We aligned the Txnip promoter sequences and found that the two ChoRE sequences, a CCAAT box, an inverted CCAAT box and a forkhead box O (FOXO) binding site were all well conserved among these species (Figure S2). We built a phylogenic tree based on the similarity of sequences covering the two ChoREs, and the tree fitted well with the evolutionary tree (fish→amphibian→mammals; Figure S3). In fish (fugu, tetraodon, zebrafish and medaka) Txnip promoters, the nucleotide sequences at the ChoRE-a position actually deviate from a canonical ChoRE; on the contrary, the fish ChoRE-b better mimics a canonical ChoRE sequence than does human ChoRE-b (Figure S4). The sequences of both the ChoRE sites on the frog Txnip promoter are moderately degenerate thus lying in between the fishes and mammals (Figure S2, S3, and S4).

### The MondoA/MLX Complex Binds to Both ChoREs In Vitro

**Figure 3:**
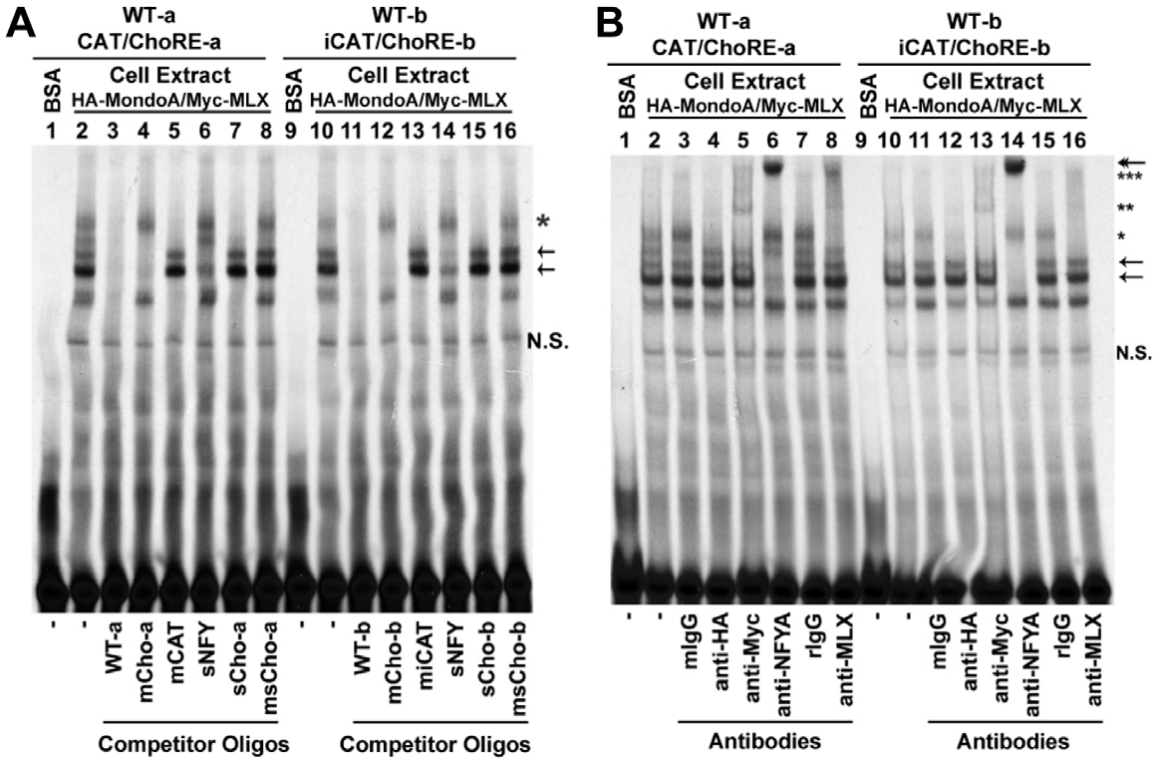
EMSAs using two Txnip promoter segments containing ChoRE-a or ChoRE-b. (A) the formation of DNA-protein complexes were competed with different cold probes. (B) the effect of different antibodies on the migration of DNA-protein complexes. Refer to text for details. N.S., non-specific.

It was shown previously that the earlier defined ChoRE (i.e., ChoRE-a) recruited a protein complex of MondoA and MLX; thus we sought to know whether the newly identified ChoRE (ChoRE-b) could also function as an anchor for MondoA and MLX. To this end, we performed electrophoresis mobility shift assays (EMSAs; Figure 3) using ^33^P-end-labeled probes and whole cell extract prepared from cells over-expressing ectopic HA-MondoA and Myc-MLX. Similar to a probe containing the ChoRE-a and CCAAT box (Figure 3A, lane 2), a probe containing the ChoRE-b and inverted CCAAT box formed multiple DNA-protein complexes (Figure 3A, lane 10); the formation of these complexes were significantly reduced by molar excess of unlabeled probes (WT; lanes 3 and 11). When molar excess of unlabeled probes of mutated ChoRE sites were used, two bands were preserved (lanes 4 and 12); the higher band (star) contained MondoA and MLX (see below), and the lower one might be due to an unknown ChoRE- or E-box-binding protein(s). The two bands were competed by shorter cold probes containing ChoREs but lacking the CCAAT boxes (sCho-a or sCho-b; lanes 7 and 15) but not by similar probes containing mutated ChoREs (msCho-a or msCho-b; lanes 8 and 16). When cold probes with mutations at the CCAAT box (mCAT) or the inverted CCAAT box (miCAT) were used in competition assays, two bands were preserved (arrows, lanes 5 and 13); these two bands were significantly competed by a short cold probe containing ATTGG sequences (sNFY, lanes 6 and 14). These results suggest that both labeled probes (CAT/ChoRE-a and iCAT/ChoRE-b) can at least form three major DNA-protein complexes, one (star) is formed between ChoRE and the MondoA/MLX complex, and the other two (arrows) are formed between CCAAT boxes (CCAAT or inverted CCAAT) and their associated factors.

To obtain identities of the protein(s) present in these complexes, we included antibodies in EMSAs (Figure 3B). The band corresponding to ChoRE (single star) was abolished by anti-HA antibodies (lanes 4 and 12), and super-shifted by anti-Myc (lanes 5 and 13; double stars) or anti-MLX (lanes 8 and 16; triple stars) antibodies. The bands corresponding to CCAAT or inverted CCAAT boxes (arrows) were super-shifted by anti-NF-YA antibodies (lanes 6 and 14; double head arrow). Naive mouse or rabbit IgG (mIgG or rIgG) as a control did not change the EMSA patterns. We conclude that HA-MondoA and Myc-MLX are able to interact with both ChoRE-a and ChoRE-b, and that nuclear factor Y (NF-Y) complex can bind to both the CCAAT and inverted CCAAT boxes, on the Txnip promoter.

### Both ChoREs Are Required for Optimal Txnip Promoter Activity

**Figure 4:**
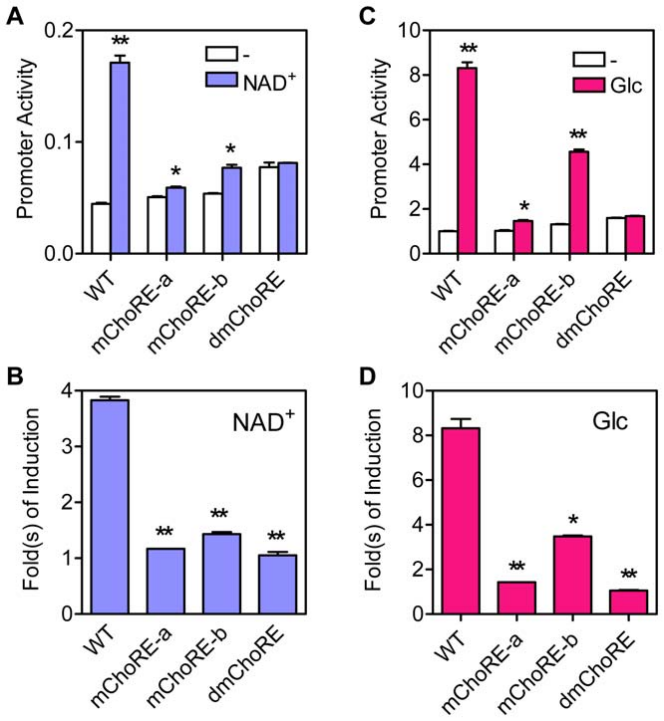
Responses of wild-type (WT) or ChoRE mutant Txnip promoters to NAD^+^ or glucose. (A and B) the effect of NAD^+^, in U2OS cells. (C and D) the effect of glucose, in L6 cells. Asterisk: significantly induced by NAD^+^ (A) or glucose (C); significantly different from wild-type promoter (B and D).

To confirm whether both ChoREs are involved in the induction of Txnip expression by glucose or adenosine-containing molecules, we have generated reporter genes driven by Txnip promoters containing mutations at ChoRE-a [37], ChoRE-b or both (dmChoRE, refer to Figure 2C), and tested their response to adenosine-containing molecules or glucose. As shown (Figure 4A and 4B), when either ChoRE or both ChoREs were mutated, the reporter genes were no longer induced by NAD^+^. For the glucose response test, the activities of promoters with mutated ChoRE-a or with double mutations at both ChoREs were not stimulated by glucose; and as compared with the wild type promoter, the response of the ChoRE-b mutant promoter was significantly reduced (Figure 4C and 4D). These results suggest that both ChoREs are required for optimal induction of Txnip expression by adenosine-containing molecules or glucose.

Given that the mChoRE-a mutant Txnip promoter possesses a more dramatically reduced response to glucose than the mChoRE-b mutant counterpart, ChoRE-a could play a major role in glucose sensing (also see discussion). On the other hand, responses of these two promoters to NAD^+^ were similar in U2OS cells. This may be due to a unique feature of the cell line used in the assay system. Indeed, for NAD^+^ response, the reduced response of the mChoRE-a mutant Txnip promoter was to a larger degree than that of the mChoRE-b mutant counterpart in HeLa cells (data not shown; also see Figure 1). Thus, in general, ChoRE-a may have higher efficacies than ChoRE-b in sensing extracellular glucose or adenosine-containing molecules.

**Figure 5:**
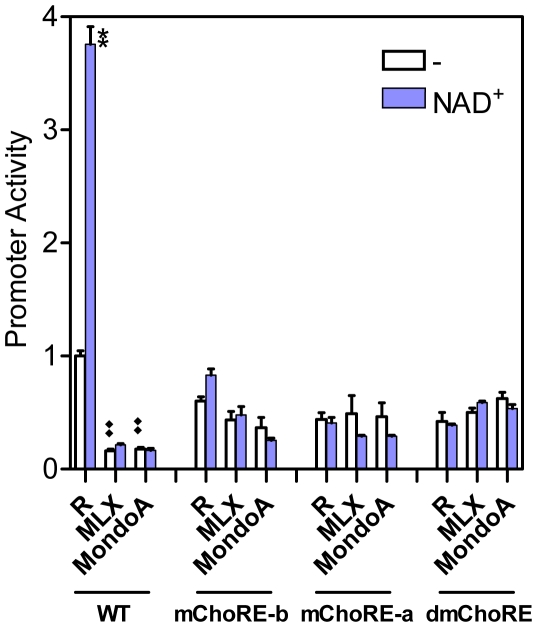
The effect of siRNAs against MLX or MondoA on Txnip promoter activity. siRNAs against MLX or MondoA down-regulated the wild-type but not the ChoRE mutant Txnip promoters and abolished the stimulatory effect of NAD^+^ on Txnip promoters in U2OS cells. Expression levels of MondoA or MLX were knocked down by specific siRNAs as shown earlier [37]. R: random (control) siRNA. Asterisk: comparison between NAD^+^ treated and untreated samples; diamond: comparison between basal activities (without NAD^+^); n = 2.

Previously, we have shown that knock-down of MondoA or MLX expression by small interfering RNAs (siRNAs) in U2OS cells reduced the basal activity of the Txnip promoter and abolished the induction by adenosine-containing molecules; for a promoter with mutation at ChoRE-a, the effect of MondoA or MLX siRNAs was diminished [37]. In this study, we found that the activities of promoters with mutations at either ChoRE or both ChoREs were largely unaffected by siRNAs targeting MondoA or MLX (Figure 5). Thus, both the ChoRE motifs are required for induction of Txnip promoter by adenosine-containing molecules, which act most likely in a MondoA/MLX dependent manner.

### ChoREs Are Not Sufficient for the Induction of Txnip Expression

**Figure 6:**
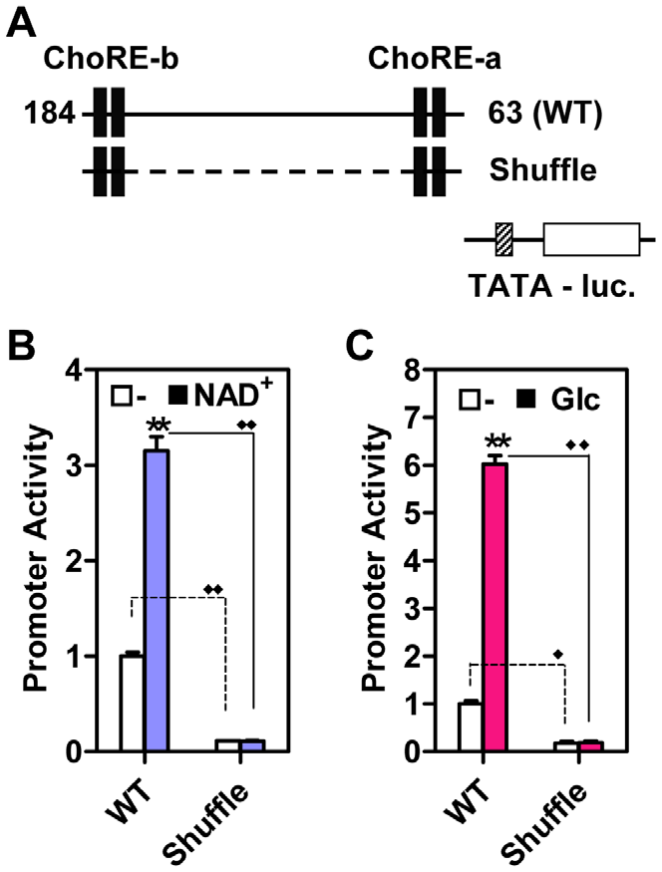
Two ChoREs are not sufficient for mediating stimulatory effect of NAD^+^ or glucose. (A) a representation of Wild-type (WT) Txnip promoter or Txnip promoter with scrambled nucleotide sequences between two ChoREs (Shuflle) used in (B) and (C). (B) the effect of NAD^+^ on Txnip promoters in U2OS cells. (C) the effect of glucose on Txnip promoters in L6 cells. Asterisk: comparison between untreated samples and samples treated with NAD^+^ or glucose; diamond: comparison between promoter activities of WT or Shuffle Txnip promoters; n = 2.

Both ChoREs are required for maximally inducing the Txnip expression by adenosine-containing molecules or glucose (Figure 4), and the promoter sequences spanning from −184 to −63 appear to contain all the information necessary to mediate the induction (Figure 2); however, we did not exclude potential contribution(s) of other sequences lying between the two ChoREs, thus asking whether the two motifs are sufficient for this stimulatory process. We hence shuffled nucleotide sequences between the two ChoREs (Shuffle, Figure 6A); the basal activity of this mutant promoter was dramatically decreased, and the promoter no longer responded to NAD^+^ (Figure 6B) or glucose (Figure 6C). We engineered tandem ChoRE-a or ChoRE-b (dChoRE-a or dChoRE-b) into the TATA box only luciferase reporter construct (Figure S5A); the activities of these chimerical promoters were not induced by NAD^+^ or Glucose (Figure S5B and S5C). Hence, the two ChoREs are not sufficient for optimal induction of the Txnip expression by adenosine-containing molecules or glucose, and additional regulatory elements are required.

### Tandem NF-Y Binding Sites Are Required for the Induction of Txnip Expression

The nucleotide sequences between two ChoREs contain a CCAAT box, an inverted CCAAT box and a FOXO binding site (Figure 2C); these three sites are highly conserved in Txnip promoters from different species (Figure S2). In EMSAs, bands corresponding to the CCAAT box or inverted CCAAT box were super-shifted by NF-YA antibodies (Figure 3B), suggesting that both the CCAAT and the inverted CCAAT boxes are potential binding sites for NF-Y.

**Figure 7:**
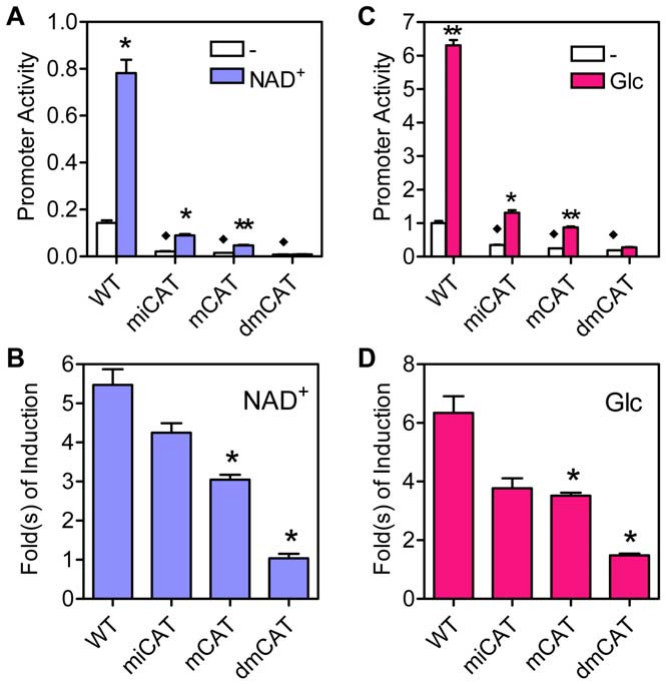
Responses of wild-type (WT) or CCAAT box mutant Txnip promoters to NAD^+^ or glucose. (A and B) the effect of NAD^+^, in U2OS cells. (C and D) the effect of glucose, in L6 cells. Asterisk: significantly induced by NAD^+^ (A) or glucose (C); significantly different from wild-type promoter (B and D); diamond: basal promoter activities were significantly different from WT promoter; n = 2.

It was previously shown that mutated FOXO binding site did not have significant effect on basal or NAD^+^-induced Txnip promoter activity, and that mutation at the inverted CCAAT box led to decreased basal activity but the responsiveness to NAD^+^ was largely intact ([37]; also confirmed in Figure 7A and 7B). In this study, the CCAAT box was also mutated (refer to Figure 2C), and the mutant promoter behaved similarly as the inverted CCAAT box mutant but to a larger degree; the promoter with mutations at both CCAAT boxes was found to have a diminished basal activity as well as an impeded induction by NAD^+^ (Figure 7A and 7B). We also examined effects of glucose on these promoters; promoters with single mutations showed retarded glucose response, and the double mutant promoter exhibited marginal response (Figure 7C and 7D). In the promoter construct with shuffled nucleotides between two ChoREs (Figure 6A), we inserted CCAAT boxes at same positions as wild type Txnip promoter (Sh-CAT, Figure S5A), and found that the activity of this promoter was induced by glucose (not NAD^+^) for ∼2-fold and fully responsive to 2-deoxy-glucose (2DG; Figure S5B and S5C), which is a glucose analog that can massively mobilize MondoA and induce Txnip expression [16]. Hence, NF-Y binding sites are not only important for maintaining the basal level transcription, but also required for the induction of Txnip expression by glucose; however, we do not rule out the possibility that additional factors other than NF-Y and MondoA/MLX, and their binding sites, might also be present for mediating an optimal response of Txnip promoter to glucose and/or adenosine-containing molecules.

### Txnip Promoter Recruits MondoA/MLX Complex in an NF-Y Dependent Manner

**Figure 8:**
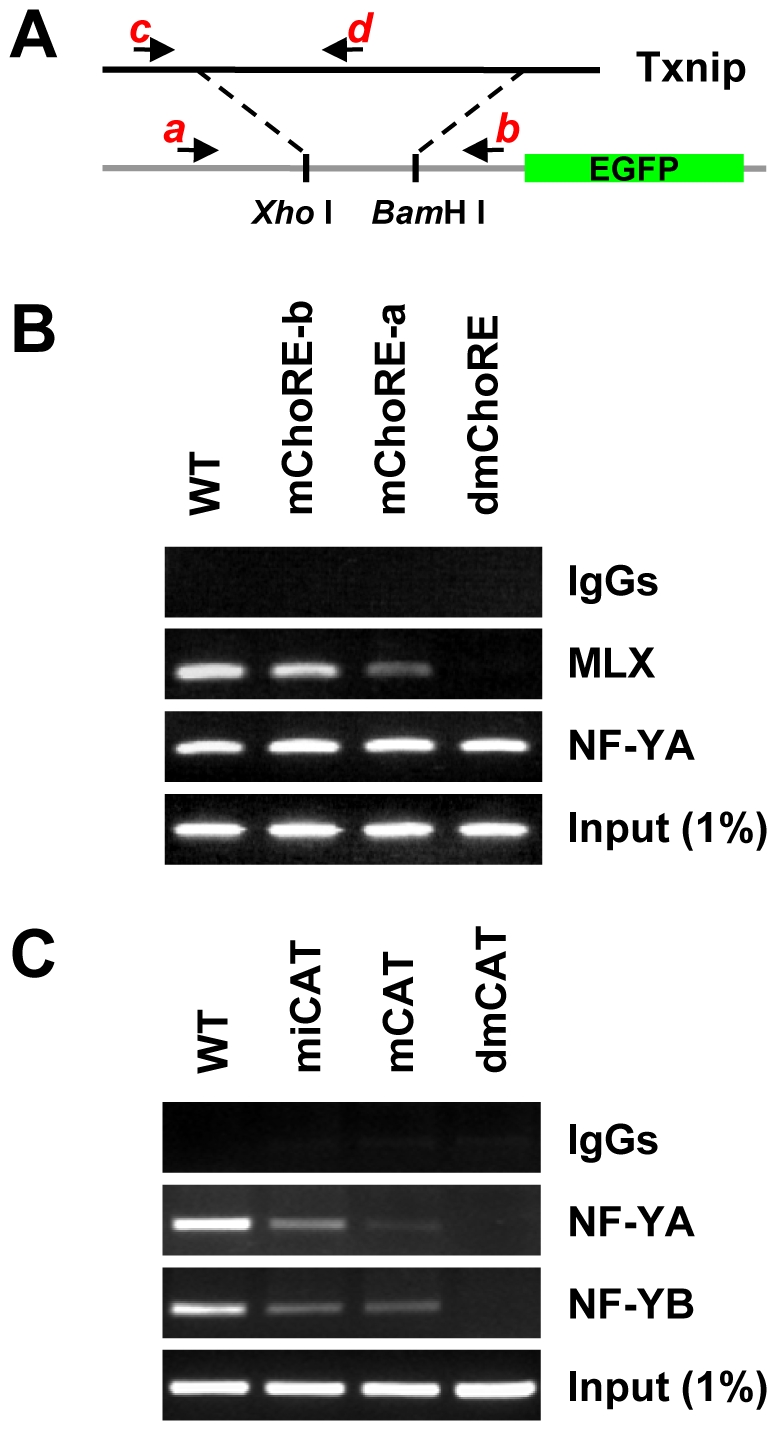
ChIP assays. (A) Txnip promoters (wild-type or mutant) were inserted into pEGFP-1 vectors using Xho I and BamH I sites, and these constructs were used to generate G418-resistant stable cell lines, which were used for ChIP assays. While primers a and b amplify the chromosomally-integrated foreign Txnip promoters, primers c and d amplify the endogenous Txnip promoter. (B) both ChoREs were able to recruit the MondoA/MLX complex. (C) both CCAAT boxes were able to recruit NF-Y.

Results from promoter assays suggest that both NF-Y and MondoA/MLX complex are required for a maximal induction of Txnip expression by glucose or adenosine-containing molecules. To confirm this, we performed chromatin immuno-precipitation (ChIP) assays to determine the occupancy of NF-Y and MLX on wild-type or mutant Txnip promoters that were parts of integrated trans-genes in stable cell lines (see “Materials and Methods” and Figure 8A for details). MLX was recruited normally to the wild type promoter, efficiently recruited to the ChoRE-b mutant promoter, and recruited to the ChoRE-a mutant promoter (albeit with a reduced efficacy); when both ChoREs were mutated, the MLX recruitment was completely impeded (Figure 8B). These results suggest that both ChoREs are able to recruit the MondoA/MLX complex, with the proximal ChoRE (ChoRE-a) being a predominant binding site. This might be in line with the severities of the mutations in the promoter functional analyses, especially regarding the glucose response (Figure 4).

In ChIP assays, the wild-type Txnip promoter was precipitated by both NF-YA and NF-YB antibodies; Txnip promoters with single mutation at the CCAAT box or the inverted CCAAT box were also precipitated by NF-Y antibodies although the signals were reduced; however, the Txnip promoter with mutations at both CCAAT boxes was not pulled-down by NF-Y antibodies (Figure 8C). This confirms that both CCAAT boxes on Txnip promoter are targets of NF-Y, and both sites are required for optimal recruitment of NF-Y onto Txnip promoter.

**Figure 9:**
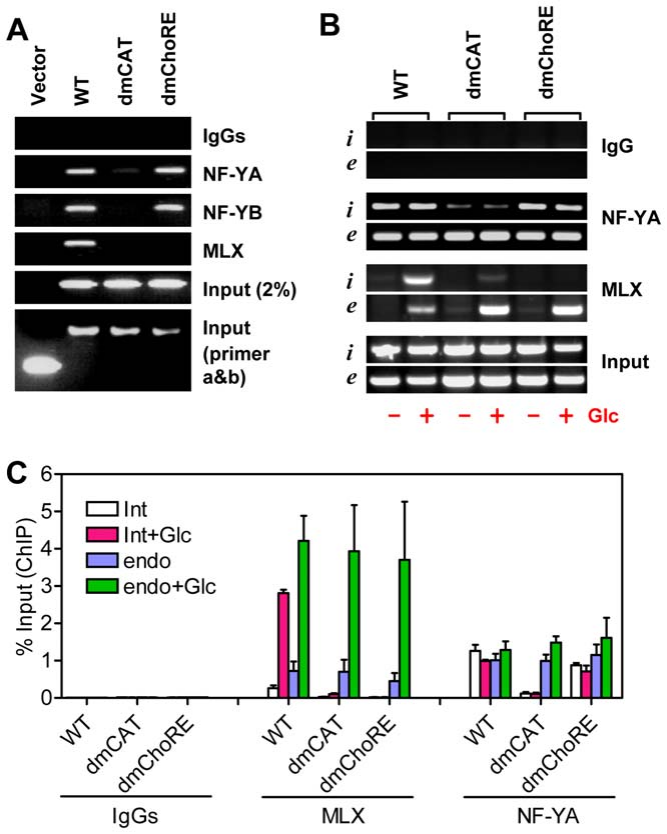
ChIP assays. (A) the occupancy of MondoA/MLX on Txnip promoter is contingent on the occupancy of NF-Y; cells were incubated in complete DMEM with 1 g/L of glucose. (B) the occupancies of MLX or NF-YA on Txnip promoters (i: integrated WT, dmChoRE or dmCAT Txnip promoter; e: endogenous Txnip promoter) in cells maintained in glucose-free medium supplemented without or with Glcuose (10 mM, 1 hrs). (C) quantification (using Real-Time PCR) for (B).

NF-YA and NF-YB were recruited onto the double ChoRE mutant (dmChoRE) promoters with a similar efficacy as the wild-type promoter (Figure 9A). Thus the interaction of NF-Y with CCAAT boxes is not dependent on ChoREs and their associated factors. Interestingly, the interaction of MLX with the double CCAAT boxes mutated promoter (dmCAT) was dramatically reduced (Figure 9A). As controls, the occupancy of MLX or NF-Y on endogenous Txnip promoters was similar in these cell lines; and MLX and NF-Y did not interact with DNA sequences located on a chromosome separated from the endogenous Txnip gene (Figure S6).

The above experiments were performed using cells maintained in complete DMEM with 1 g/L glucose; we also tested the Txnip promoter occupancies by MLX or NF-Y in cells incubated in glucose-free medium supplemented without or with glucose (10 mM). As shown (Figure 9B and 9C), NF-YA was recruited to the integrated (i) wild type or ChoRE-mutated (dmChoRE) promoters or the endogenous (e) Txnip promoter at similar efficacies under both the glucose-free and glucose-treated conditions, but not to the double CCAAT box-mutated (dmCAT) Txnip promoter, suggesting that the CCAAT box binding by NF-Y was not sensitive to glucose. On the other hand, under glucose-free condition, only residue amount of MLX was bound with the Txnip promoters (WT or dmCAT) and, in cells treated with glucose, the MLX recruitment to the integrated wild type and endogenous Txnip promoters was dramatically increased; however, the MLX recruitment to the dmCAT promoter was significantly repressed. The occupancies of the integrated wild type promoter by MLX or NF-Y were similar to that of the endogenous Txnip promoter in stable cell lines, suggesting that a chromosomally-integrated transfected promoter can function similarly as the endogenous counterpart. Thus, mutations at the CCAAT boxes can effectively disrupt the interaction of cognate factors with the Txnip promoter, and the recruitment of NF-Y to Txnip promoter is probably a prerequisite for the recruitment of MondoA/MLX complex.

**Figure 10:**
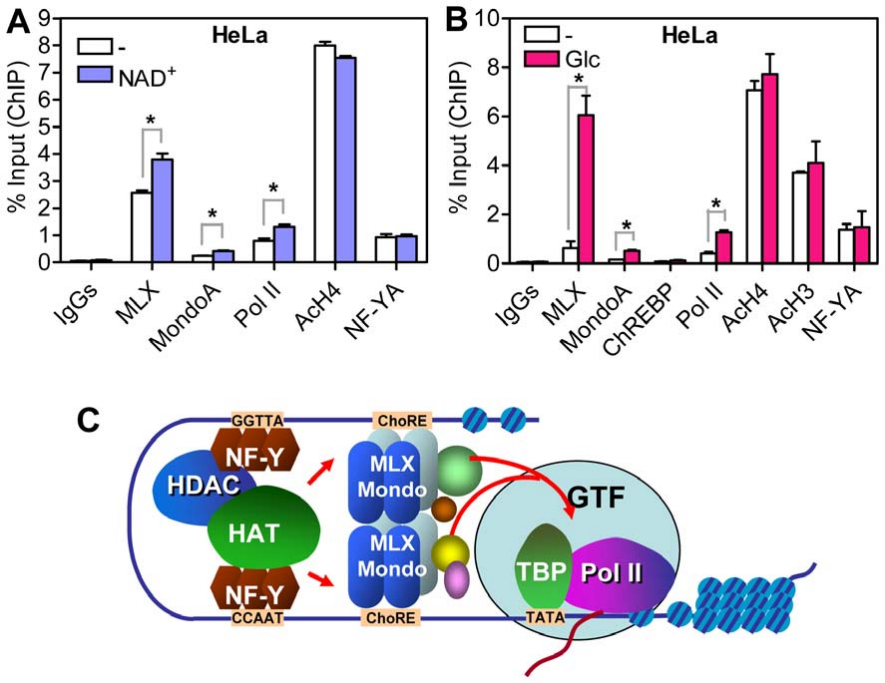
ChIP asssys and a working model. The occupancy of MLX, MondoA, ChREBP, Pol II or NF-YA on the Txnip promoter in HeLa cells under NAD^+^ (0.1 mM for 4 hrs; A) or Glucose (10 mM for 1 hr; B) treatment; the acetylation status of Txnip promoter associated H3 and H4 under different conditions was also examined. Asterisk: significantly induced by NAD^+^ or glucose. (C) a model for the transcriptional regulation of the Txnip gene promoter by NF-Y, MLX, Mondo (MondoA or ChREBP) and other (co)factors. See text for further details.

We also performed ChIP assays using different cells treated with or without glucose or NAD^+^. In HeLa cells, the occupancies of MLX, MondoA and RNA polymerase II (Pol II) on Txnip promoter were significantly increased under NAD^+^ or glucose treatment (Figure 10A and 10B). Histone proteins around Txnip promoter was highly acetylated, even in cells incubated in glucose-free medium for 16 hrs (Figure 10B), which suggests that the Txnip gene promoter is relatively open at a non-induced state. We also found that the interaction of ChREBP (a MondoA homolog, also known as MondoB) with Txnip promoter was not detected in HeLa cells (Figure 10B). Similar results were obtained when U2OS or L6 cells were used (Figure S7A and S7B). On the other hand, in INS-1 (pancreatic) or HepG2 (hepatic) cells, ChREBP, rather than MondoA, occupies the Txnip promoter in a glucose-inducible manner (Figure S7C and S7D). This is in line with high ChREBP expression levels in these cell types and suggests that either ChREBP or MondoA, in a tissue-selective way, may function with MLX to support glucose induced Txnip expression. Consistent with a potent response to NAD^+^ [37], recruitment of MLX, MondoA and Pol II to the Txnip promoter was increased under NAD^+^ treatment in U2OS cells (Figure S7E). In all tested cell lines, the recruitment of NF-YA to the Txnip promoter was largely constant, suggesting a role of NF-Y complex in maintaining the basal transcription level of the Txnip gene.

## Discussion

### Tandem ChoREs on the Txnip Gene Promoter

It has been shown that the induction of Txnip expression by glucose or adenosine-containing molecules was mediated by the proximal ChoRE and its associated transcription factors MondoA/MLX [16], [22], [37]. When testing responses of a series of truncated or mutated Txnip promoters to NAD^+^ or glucose (Figures 1 and S1), we found that the sequence around 170 bp upstream of the transcription start site was also critical for the induction; this sequence is highly conserved in the Txnip promoters of diverse species and resembles a degenerate ChoRE (Figures 2, S2-S4). In EMSA and reporter assays, this newly identified ChoRE, designated ChoRE-b, functioned similarly as the previously reported ChoRE (ChoRE-a; Figures 3 and 4). In ChIP assays, when ChoRE-a was mutated, MLX could be recruited onto the Txnip promoter presumably via a weak interaction with ChoRE-b (Figure 8B). Thus, we conclude that there are two ChoREs on the Txnip promoter, in which the ChoRE-b is also a significant contributor to the induction of Txnip expression by glucose or adenosine-containing molecules in a MondoA/MLX dependent fashion.

A recent study by other colleagues paid attention to the Txnip promoter region around ChoRE-b as well, and concluded that a potential ChoRE (GAGCAC ACCGTGTCCACGCG) was not critical for the stimulatory effect of glucose on Txnip expression [39]. Our view is that the ChoRE-b nucleotide sequences should be GAGCACACCGTGTCCACGCG , especially when the fish Txnip promoters were considered (Figure S4). Therefore, the results and the conclusion in their study might be due to site-directed mutagenesis that was off-target, or due to employment of cells that were less sensitive to a missing function of ChoRE-b and the associated factors. In our hands, the response of Txnip promoters lacking a functional ChoRE-b to glucose treatment was significantly reduced, which was observed in various tested cell lines such as L6 (Figure 1B), HepG2, HeLa and C2C12 cells (data not shown).

We realized, however, that the activities of ectopic Txnip promoters lacking a functional ChoRE-b were induced by NAD^+^ in HeLa cells but not in U2OS cells (Figure 1A); this may suggest that the epigenetic statuses of the Txnip promoter may vary in different cell types. The action of adenosine-containing molecules is on top of the glucose-induced Txnip expression i.e., amplification of the glucose signaling [37]; based on the integrity of glucose signaling, the Txnip promoter in U2OS cells may have a more open chromatin structure than that in HeLa cells. This may explain the more dramatic induction of the Txnip expression in U2OS cells than that in HeLa and other cells in responding to NAD^+^ or ATP [37]. In this scenario, the Txnip promoter in U2OS cells could be more sensitive to missing functions of a ChoRE-b element and associated MondoA/MLX. Alternatively, and supported by preliminary results (data not shown), signaling pathway(s) evoked by the adenosine-containing molecules in U2OS cells could be unique and be different from those evoked in HeLa or other cell types. Nevertheless, the glucose response, or the response to adenosine-containing molecules, of the Txnip promoter is a ubiquitous phenomenon.

### Full Induction Requires Both ChoREs and CCAAT Boxes

Within the minimal promoter (184–63) that supported full induction (Figure 2B), two ChoREs, two CCAAT boxes and one FOXO-binding site have been identified (Figure 2C). Among these motifs, the FOXO-binding site is probably required for mediating the inhibition of Txnip expression by insulin [20], [25], [40]; the proximal ChoRE (ChoRE-a) has been shown to be critical for the induction of Txnip expression by glucose or adenosine-containing molecules [22], [37]; and the inverted CCAAT box was identified as an element responsible for mediating the stimulatory effect of SAHA [13].

Here, however, we have found that both the distal (ChoRE-b) and the proximal (ChoRE-a) ChoREs on Txnip promoter are required for the induction of Txnip expression by glucose or adenosine-containing molecules. It is possible that, for optimal induction of Txnip expression, the two ChoREs might well be organized in proximity by the interplay of their associated transcription factors (MLX/MondoA) and cognate co-activators to stimulate Txnip expression (Figure 10C). This is similar to a model proposed for transcription stimulation of the MCP-1 gene, in which two tandem nuclear factor κB (NF-κB) binding sites recruit NF-κB proteins and co-activators p300/CBP upon TNF or LPS stimulation [41].

Two tandem ChoREs alone, however, are not sufficient, and two tandem NF-Y-binding sites, i.e., the CCAAT and the inverted CCAAT motifs, are additionally required for optimal Txnip expression induction by NAD^+^ or glucose (Figures 6 and 7). Txnip promoters with a singly mutated CCAAT box exhibited much reduced activities but nevertheless retained the induction potentials by NAD^+^ or glucose; however, when both CCAAT motifs were mutated, the induction potentials were completely abolished (Figure 7). We propose that the NF-Y-binding sites are not only critical for maintaining a basal Txnip promoter activity but also involved in the induction of Txnip expression by glucose or adenosine-containing molecules, presumably through sustaining certain chromatin status that helps optimize the induction (Figure 10C; and also see below).

### NF-Y and MondoA/MLX Cooperate to Stimulate Txnip Expression

The NF-Y is a complex containing NF-YA, NF-YB and NF-YC subunits, all of which are required for interaction with the CCAAT motif that is a widely distributed promoter element in human genome [42]. NF-Y can recruit certain histone acetyltransferases (HATs) such as GCN5 and p300/CBP-associated factor (PCAF) onto a target promoter, adding acetylation marks onto histones and transcription factors [43]. These epigenetic marks usually positively impact target gene transcription (reviewed in [44]). Consistent with this notion, the inverted CCAAT box is known to mediate the Txnip expression induction by SAHA [13], which as a histone deacetylase inhibitor can in principle counter-act the removal of the above epigenetic marks hence sustaining gene activation.

Our ChIP results indicate that NF-Y was recruited onto the Txnip promoter via both CCAAT boxes (Figure 8C); this promoter occupation seems to be a prerequisite for the recruitment of the MondoA/MLX complex (Figure 9). Hence, NF-Y and MondoA/MLX might coordinate to maximize the Txnip expression. NF-Y constitutively occupies the Txnip promoter even in glucose-free medium, and this binding might facilitate the Txnip promoter occupancy, in a glucose-dependent manner, by MondoA/MLX. These promoter-bound factors in turn recruit co-activator(s), which set up local epigenetic environment that favors the induction by other signals from, e.g., adenosine-containing molecules (Figure 10C).

A very recent paper has reported the Txnip promoter dynamics under glucose treatment in INS-1 cells, of pancreatic origin, using ChIP assays [45]. This study shows that, with glucose stimulation, ChREBP can recruit p300 to increase histone acetylation, which in turn enhances recruitment of Pol II to induce the Txnip transcription. Indeed, we found that ChREBP, but not MondoA, was recruited to the Txnip promoter in (pancreatic) INS-1 cells and (hepatic) HepG2 cells (Figure S7C and S7D). In our hands, these cell lines express high ChREBP levels, as opposed to low levels of MondoA (not shown). Thus, in a cell-context-dependent manner, either MondoA or ChREBP may respond to glucose evoked signaling and induce Txnip expression (Figure 10C; here, Mondo represents either MondoA or ChREBP); however, we do not rule out the possibility that transmitting the glucose signaling to MondoA or ChREBP (in complex with MLX) might involve distinct intermediate molecules.

It has been shown that NF-Y can also cooperate with several other transcription factors, such as upstream stimulatory factors (USF1/2), Oct1, GATA-1, SP-1, ATF-2, Regulatory factor X (RFX) and sterol regulatory element binding protein-1 (SREBP1) [46]–[52] to regulate the transcription of target genes under nutritional, hormonal or immunological challenges. For instance, the promoter of another metabolic related gene, that of the type II Hexokinase (HKII), contains a CCAAT box, an inverted CCAAT box and multiple GC boxes that are bound by the SP family transcription factors [53]. CCAAT box-containing promoters might employ a common regulatory mechanism in which NF-Y might function as a regulator that facilitates the function of other gene specific transcription factors to induce gene transcription as illustrated in Figure 10C. On the more distal Txnip promoter region, there is a vitamin D response element and a glucocorticoid response element [13], [30]; it would be of high interest to study if NF-Y can also cooperate with vitamin D or glucocorticoid receptor to mediate vitamin D or glucocorticoid induced Txnip expression.

## Materials and Methods

### Cell Culture

Cells were maintained under 5% CO_2_ at 37°C in low glucose (1 g/L) DMEM supplemented with antibiotics, L-glutamine and 10% fetal bovine serum (HyClone, Logan, UT). The glucose-free DMEM medium was supplemented with (additional) 2 mM of sodium pyruvate. DMEM was from Sigma Chemical Co. (St. Louis, MO), and other supplements were from Invitrogen (Carlsbad, CA).

### RNA Extraction, RT (Reverse Transcription)-PCR, and Real-Time PCR

RNA was extracted using RNeasy Mini Kit (QIAGEN, Valencia, CA). Reverse transcription was carried out using SuperScript III reverse transcriptase and random hexamers, RNaseOUT was used to maximize RNA stability (all reagents for reverse transcription were from Invitrogen). PCR and Real-Time PCR were carried out using the Taq DNA polymerase (New England Biolabs, Ipswich, MA) and Sybr Green Core Reagents (Applied Biosystems, Foster City, CA) respectively. Same primers (synthesized by Proligo, Singapore) were used in RT-PCR and Real-Time PCR, the sequences of primers are shown in Table S1.

### Plasmid Constructs

Phusion DNA polymerase (Finnzymes, Espoo, Finland) was used in constructing Txnip promoter plasmids with deletions or mutations. From a long Txnip promoter, various promoter fragments were generated by subsequent PCR, covering 1299, 269, 169, 163, 142, 102 and 73 bp Txnip promoter sequences (to the transcription start site), and inserted into the pGL3 vector using the Xho1 and Nhe1 sites. Mutations at ChoREs or CCAAT boxes were introduced into the wild-type Txnip prompter (−269) by PCR. MondoA and MLX expression constructs were described in Yu et al. [37].

### siRNAs

The sequence information of siRNAs against MondoA or MLX was described in Yu et al. [37].

### Transfection

Cells cultured in 6-well plates (about 80% confluency) were washed with OPTI-MEM (Invitrogen) and used for transfection. During transfection, a mixture of 4 µl of lipofactamine 2000 (Invitrogen), 1 µg of Txnip promoter plasmid and 10 ng of simian virus 40 (SV40)-renilla luciferase plasmid (top up to 100 µl using OPTI-MEM) were prepared. After 20 min, the mixture was added drop-wise into the well with cells incubated in OPTI-MEM (0.9 ml). At 5 hrs, the medium was replaced with complete medium. For co-transfection of plasmids and siRNA, 5 µl of lipofactamine 2000, 1 µg of Txnip promoter plasmid, 100 pmole siRNA and 10 ng of renilla luciferase plasmid were used.

### Promoter Activity (Reporter) Assays

Cells were treated with 0.2 mM of NAD(H) or 10 mM of glucose for 16 hrs after transfection. Cell lysates were prepared and the firefly or renilla luciferase activities were measured using the Dual-Luciferase Reporter Assay System (Promega, Madison, WI).

### Electrophoresis Mobility Shift Assay (EMSA)

Oligonucleotides (synthesized by Proligo, see Table S2 for sequences information) were labeled with [α-^33^P]dATP (GE Healthcare, Bucks, UK) using Klenow enzymes (exo^−^, New England Biolabs). In a typical binding reaction, 10 fmole end-labeled oligonucleotides in 2× EMSA buffer (25 mM Hepes-KOH at pH 7.9, 62.5 mM KCl, 0.05% Nonidet P-40, 2 mM MgCl_2_, 8% Ficoll 400, 500 µg/ml BSA, and protease inhibitors) were mixed with 5 µg of whole cell extract prepared from HeLa cells over-expressing HA-MondoA and Myc-MLX; the reaction was carried out at room temperature for 30 min. For competition assays, 100× molar excess cold oligonucleotides were added; and for antibody inhibition or super-shift assays, 0.5 µg of each antibody was included in the binding reaction. Protein-DNA complexes were then separated on 4% polyacrylamide gels (in 6.25 mM Tris at pH 8.2, 50 mM Glycine, 0.1 mM EDTA, 1 mM MgCl_2_, 0.025% Nonidet P-40 and 0.5 mM DTT) and visualized by autoradiography.

### Chromatin Immunoprecipitation (ChIP)

Wild-type or mutant Txnip promoter (same as promoters used in luciferase reporter assay) was inserted into pEGFP-1 vector. These plasmid constructs and empty pEGFP-1 vector were transfected into HeLa cells. G418 resistant colonies were selected, and stable cell lines were established and used for ChIP assays. The presence of these plasmids in stable cells lines were confirmed by PCR or fluorescence microscopy. Following different treatments, cells were treated with 1% formaldehyde for 10 min at room temperature, and DNA-protein cross-linking reaction was stopped by the addition of glycine. Cells were harvested and washed with PBS twice and then incubated in cold MC buffer [54] for 30 min. Cells were then subjected to sonication and immunoprecipitation using ChIP assay kit (Millipore, Bedford, MA). A mixture of Protein A and Protein G agarose beads (1∶1 in ratio) was used for pre-clearance and pull-down of antibody-protein-DNA complexes, and DNA fragments extricated from these complexes were purified using QIAquick PCR Purification Kit (Qiagen). Finally, recovered DNA fragments of interest were analyzed by PCR using Taq DNA polymerase (New England Biolabs). Antibodies used in ChIP assays are: MLX (AF4186; R&D Systems, Minneapolis, MN), NF-YB (pAb-077-050; Diagenode, Belgium), AcH4 and AcH3 (06-598 and 06-599, Millipore), Pol II (MMS-126R; Covance, Princeton, NJ), all other antibodies (NF-YA, sc-100779; ChREBP, sc-21189; MondoA, sc-133397; and navie IgGs) are from Santa Cruz Biotechnology (Santa Cruz, CA). The information of PCR primers used is shown in Table S1.

### Statistical Analyses

Unpaired *t*-test was used for data analysis in this study, and two tailed P value less than 0.05 was considered statistically significant. P values less than 0.05 was indicated by asterisk (*) or diamond (♦), and P values less than 0.005 was indicated by double asterisks or diamonds; data without these indicators suggest P values at ≥0.05.

## Supporting Information

Figure S1 Promoter activity of Txnip promoters. (A) In HeLa cells, promoters with a 142 bp or longer Txnip promoter sequence were induced by NAD^+^. (B) In U2OS cells, promoters with a 169 bp or longer Txnip promoter sequence were induced by NAD^+^. (C) In L6 cells, promoters with a 142 bp or longer Txnip promoter sequence were induced by glucose. Asterisks indicate the promoter was significantly induced by NAD^+^ or glucose (refer to “experimental procedures” for details of statistics).
(0.39 MB JPG)

Figure S2 Alignment of Txnip promoters from different species. Sequence alignment of Txnip promoters. Txnip promoter sequences of different species were aligned using CLUSTAL W program. The conserved cis-elements (ChoREs, FOXO-binding site, CCAAT, or inverted CCAAT) were highlighted using boxes.
(0.97 MB TIF)

Figure S3 The phylogenetic tree built from Txnip promoters of different species using the Neighbor-Joining (NJ) method.
(0.33 MB JPG)

Figure S4 Sequence alignment of fish and frog Txnip promoters with human Txnip promoter. Sequences corresponding to ChoRE-a in fish Txnip promoters are not a good ChoRE (as indicated by red boxes). Sequences corresponding to ChoRE-b in fish Txnip promoters are more similar to the canonical ChoRE (green box with dotted lines).
(0.45 MB JPG)

Figure S5 The response of hybrid Txnip promoters to NAD^+^ or glucose. (A) Fusion of a TATA box-driven luciferase reporter with Txnip prompters. Shuffle, nucleotide sequences between two ChoREs were scrambled; Sh-CAT, shuffle with two CCAAT boxes; other promoters contain two ChoREs. (B) The activity of the wild-type Txnip promoter, but not the other Txnip promoters, was induced by NAD^+^. (C) The activity of Txnip promoters without CCAAT boxes was not induced by glucose. Note that the Sh-CAT promoter showed normal basal activity (not shown), which was not significantly induced by NAD^+^ (B), but was significantly and fully induced, respectively, by glucose and 2DG (C).
(0.51 MB JPG)

Figure S6 ChIP assays. (A) The endogenous Txnip promoter was precipitated in a similar fashion using different cell lines. (B) A negative control DNA was not precipitated by antibodies against MLX or NF-YB.
(0.21 MB JPG)

Figure S7 ChIP assays. The interaction of MLX, MondoA, ChREBP, Pol II, or NF-YA with Txnip promoter was analyzed in different cells under glucose (A–D) or NAD^+^ (E) treatment. The acetylation status of Txnip promoter-associated H4 was also examined.
(0.72 MB JPG)

Table S1 Sequence information of oligonucleotides.
(0.05 MB DOC)

Table S2 Sequence information of probes used in EMSA.
(0.04 MB DOC)
