# Supplementary figures and images for: Tandem ChoRE and CCAAT Motifs and Associated Factors Regulate Txnip Expression in Response to Glucose or Adenosine-Containing Molecules
Source: PLoS One. 2009 Dec 22;4(12):e8397. doi: 10.1371/journal.pone.0008397 (PMC2791861; doi:10.1371/journal.pone.0008397)

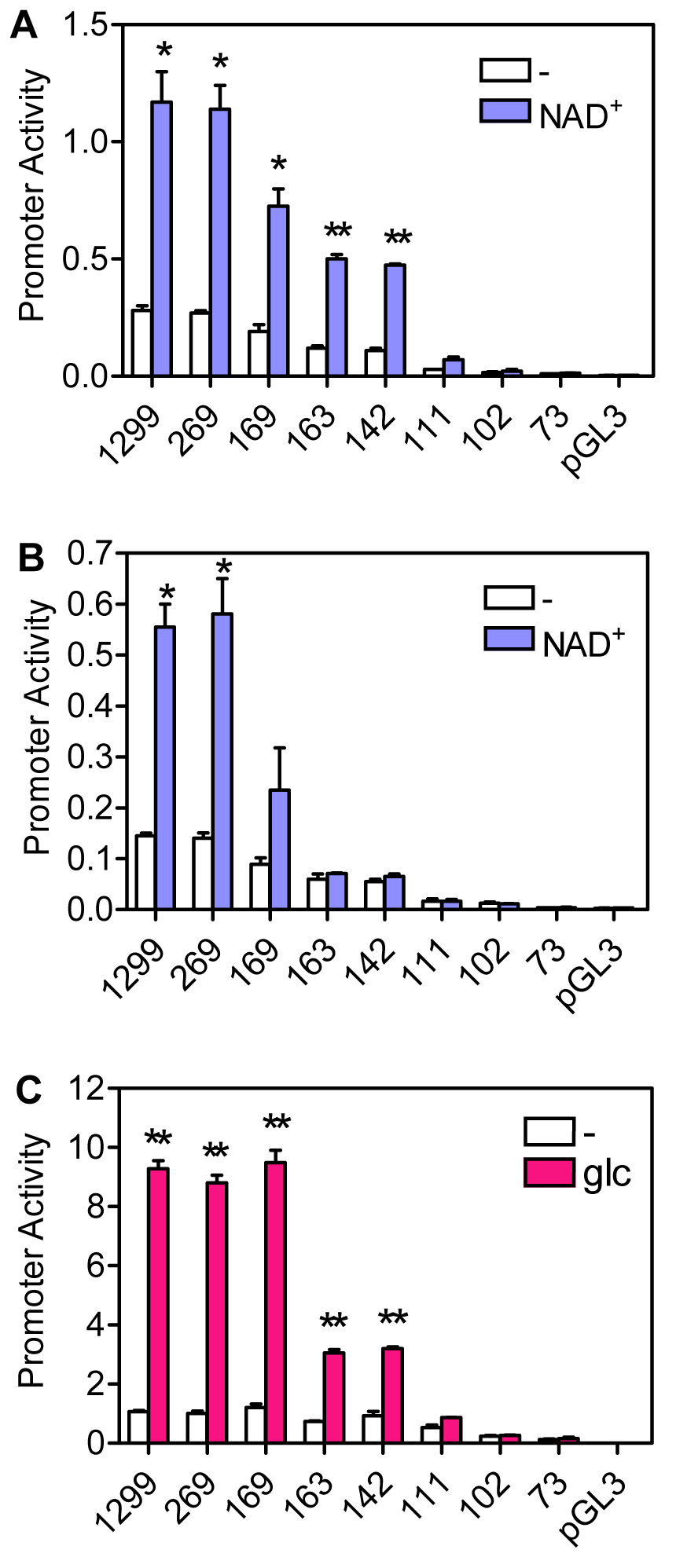

Supplement: Figure S1 — Promoter activity of Txnip promoters. (A) In HeLa cells, promoters with a 142 bp or longer Txnip promoter sequence were induced by NAD+. (B) In U2OS cells, promoters with a 169 bp or longer Txnip promoter sequence were induced by NAD+. (C) In L6 cells, promoters with a 142 bp or longer Txnip promoter sequence were induced by glucose. Asterisks indicate the promoter was significantly induced by NAD+ or glucose (refer to “experimental procedures” for details of statistics). (0.39 MB JPG) [file pone.0008397.s001.jpg]

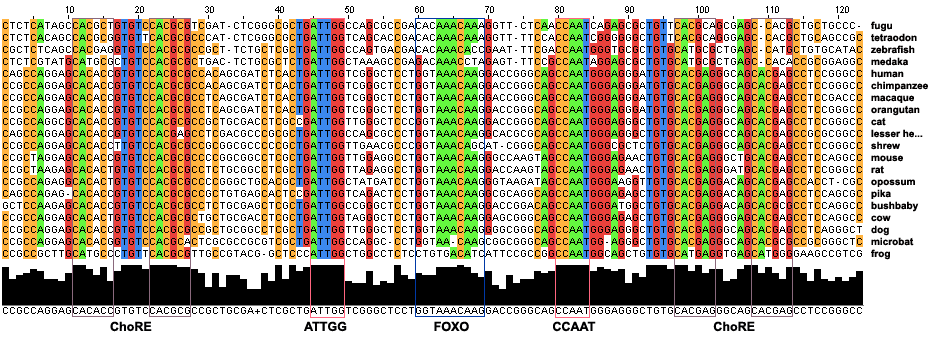

Supplement: Figure S2 — Alignment of Txnip promoters from different species. Sequence alignment of Txnip promoters. Txnip promoter sequences of different species were aligned using CLUSTAL W program. The conserved cis-elements (ChoREs, FOXO-binding site, CCAAT, or inverted CCAAT) were highlighted using boxes. (0.97 MB TIF) [file pone.0008397.s002.tif]

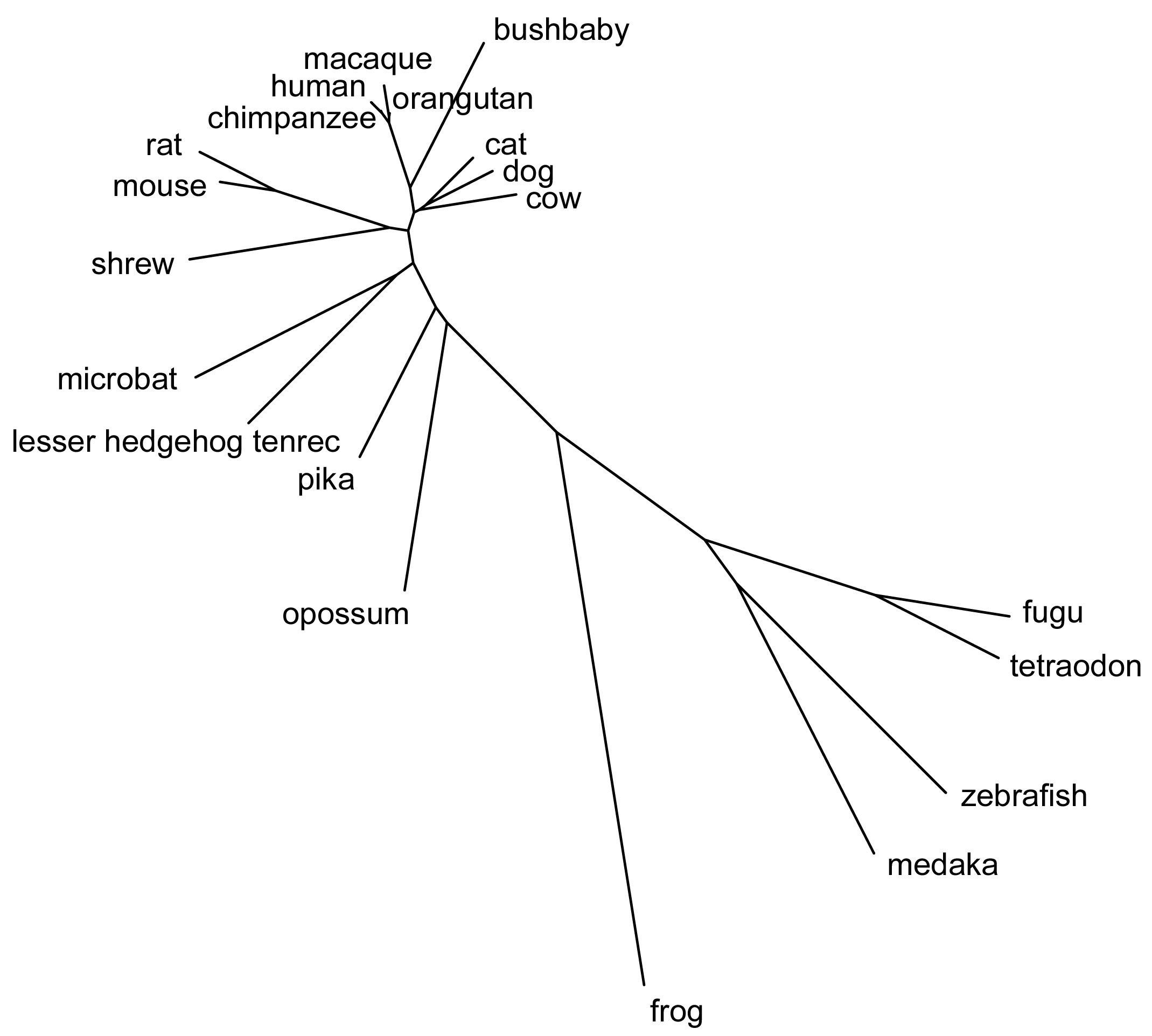

Supplement: Figure S3 — The phylogenetic tree built from Txnip promoters of different species using the Neighbor-Joining (NJ) method. (0.33 MB JPG) [file pone.0008397.s003.jpg]

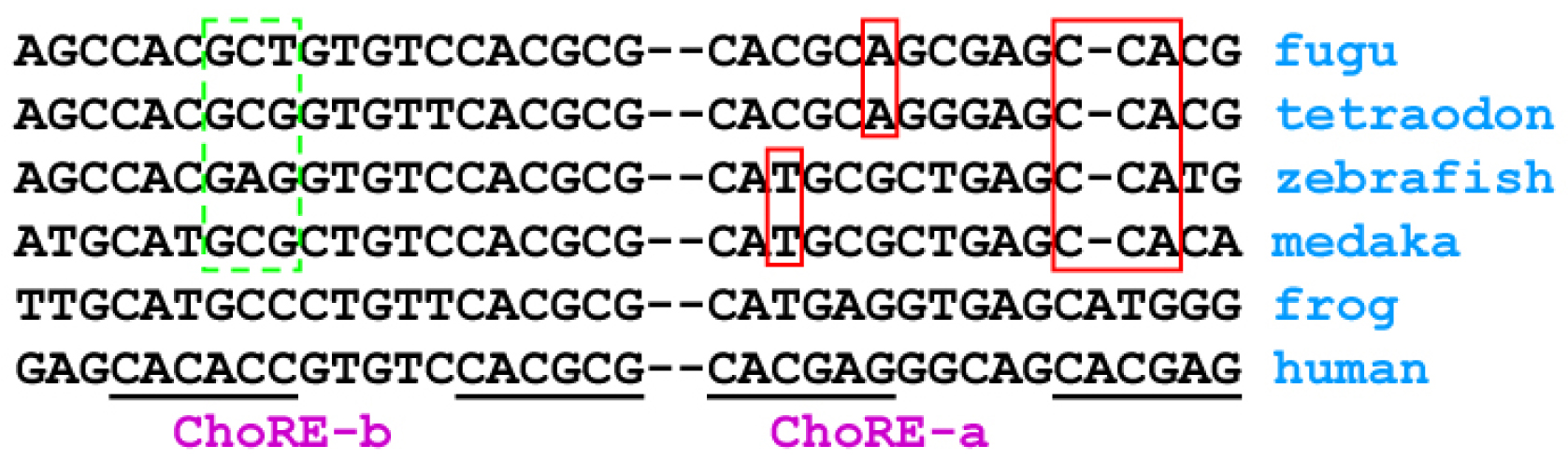

Supplement: Figure S4 — Sequence alignment of fish and frog Txnip promoters with human Txnip promoter. Sequences corresponding to ChoRE-a in fish Txnip promoters are not a good ChoRE (as indicated by red boxes). Sequences corresponding to ChoRE-b in fish Txnip promoters are more similar to the canonical ChoRE (green box with dotted lines). (0.45 MB JPG) [file pone.0008397.s004.jpg]

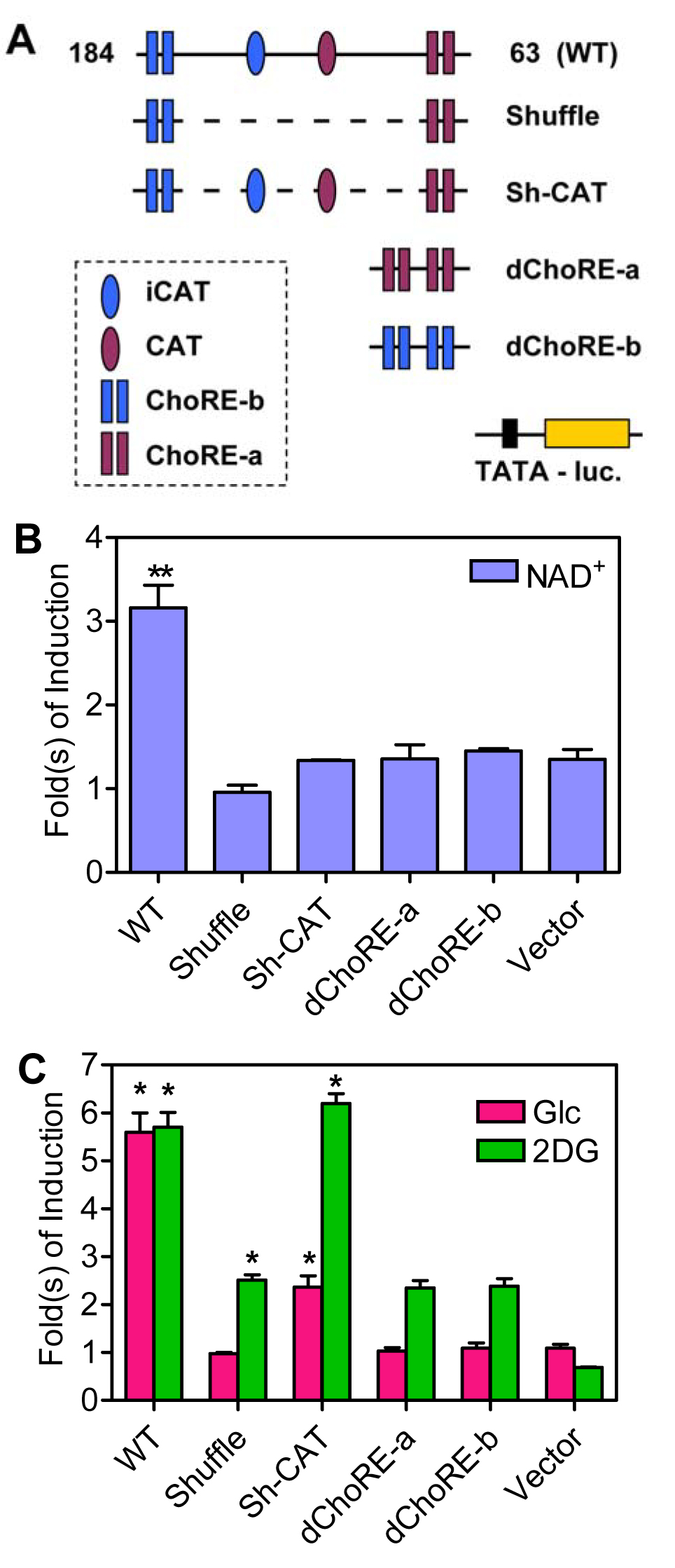

Supplement: Figure S5 — The response of hybrid Txnip promoters to NAD+ or glucose. (A) Fusion of a TATA box-driven luciferase reporter with Txnip prompters. Shuffle, nucleotide sequences between two ChoREs were scrambled; Sh-CAT, shuffle with two CCAAT boxes; other promoters contain two ChoREs. (B) The activity of the wild-type Txnip promoter, but not the other Txnip promoters, was induced by NAD+. (C) The activity of Txnip promoters without CCAAT boxes was not induced by glucose. Note that the Sh-CAT promoter showed normal basal activity (not shown), which was not significantly induced by NAD+ (B), but was significantly and fully induced, respectively, by glucose and 2DG (C). (0.51 MB JPG) [file pone.0008397.s005.jpg]

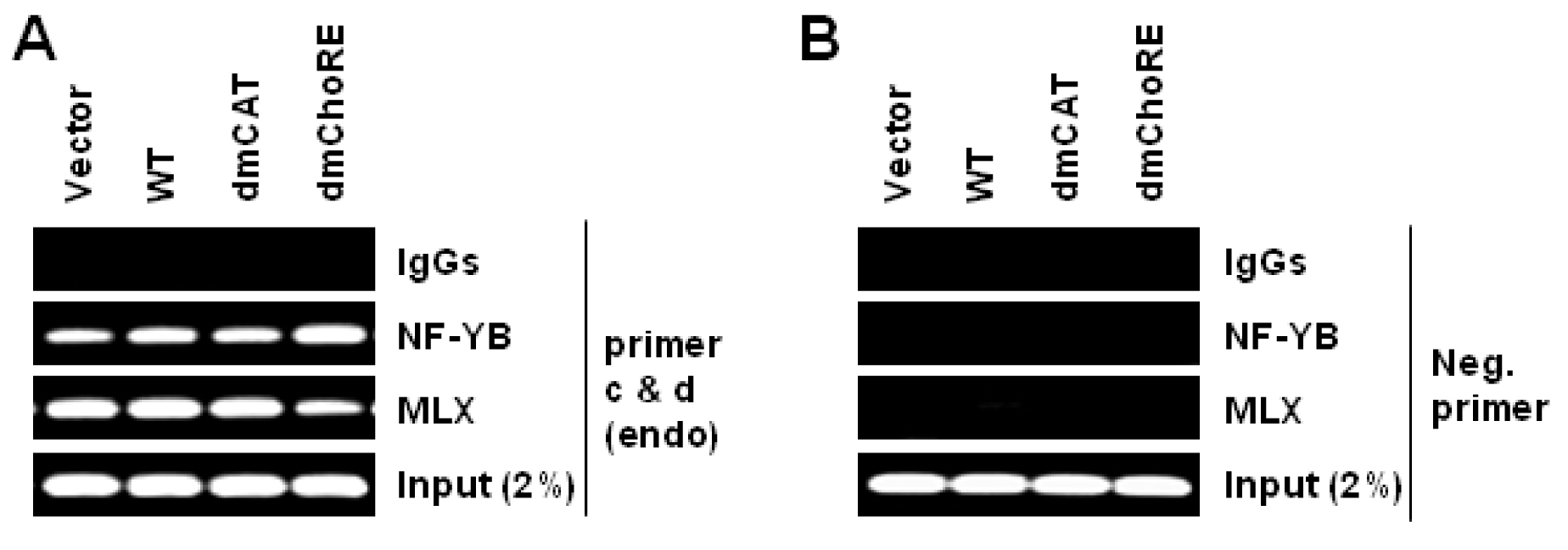

Supplement: Figure S6 — ChIP assays. (A) The endogenous Txnip promoter was precipitated in a similar fashion using different cell lines. (B) A negative control DNA was not precipitated by antibodies against MLX or NF-YB. (0.21 MB JPG) [file pone.0008397.s006.jpg]

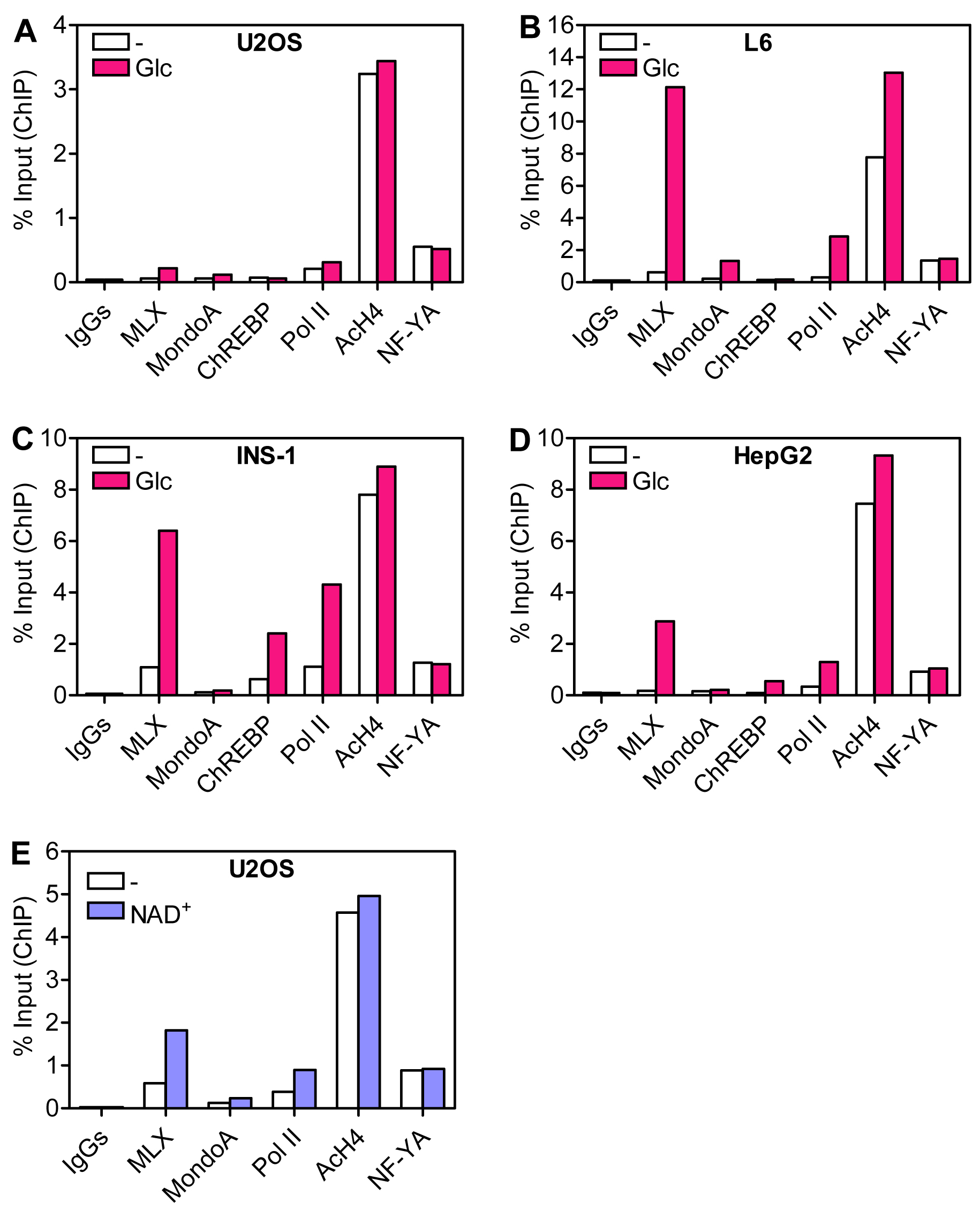

Supplement: Figure S7 — ChIP assays. The interaction of MLX, MondoA, ChREBP, Pol II, or NF-YA with Txnip promoter was analyzed in different cells under glucose (A–D) or NAD+ (E) treatment. The acetylation status of Txnip promoter-associated H4 was also examined. (0.72 MB JPG) [file pone.0008397.s007.jpg]
