# Supplementary material for: Tandem ChoRE and CCAAT Motifs and Associated Factors Regulate Txnip Expression in Response to Glucose or Adenosine-Containing Molecules
Source: PLoS One. 2009 Dec 22;4(12):e8397. doi: 10.1371/journal.pone.0008397 (PMC2791861; doi:10.1371/journal.pone.0008397)
Supplement: Table S1 — Sequence information of oligonucleotides. (0.05 MB DOC) [file pone.0008397.s008.doc]

**Table S1. Sequence Information of Oligonucleotides**

| **Oligonucleotides** | **Remarks** |
| --- | --- |
| GCCTCGAGCTCCAAATCGAGGAAACCG-reverse | Primers for generating serial Txnip promoter deletions. The reverse primer is a common. Numbers indicate the base pairs upstream of the transcription staring site. Restriction sites are underlined. |
| CCGGCTAGCCCAACAAGAATGAAGAGAG-1299 |
| CCGGCTAGCACAGCCCCCTCCTTCCC-269 |
| CCGGCTAGCGTGTCCACGCGCCACAGC-169 |
| CCGGCTAGCACGCGCCACAGCGATCTC-163 |
| CCGGCTAGCGATTGGTCGGGCTCCTGG-142 |
| CCGGCTAGCAGCCAATGGGAGGGATG-111 |
| CCGGCTAGCGGGAGGGATGTGCACGAGGG-102 |
| CCGGCTAGCCCTCCGGGCCAGCGCTCG-73 |
| CCGGCTAGCCCCTCCCATTGGCTGCCCG-Rev95 | Primers for generating Txnip-TATA fusion promoters. |
| CCGGCTAGCGGTCCGGAGGCTCGTGCTGC-Rev63 |
| CCGACGCGTACAGCCCCCTCCTTCCC-269 |
| CCGACGCGTAGCCAGGAGCACACCGTGTC-184 |
| CCGACGCGTCTGATTGGTCGGGCTCCTGG-145 |
| CGTGTCCACGCGCCACAGCGAT-d170fwd |
| CTGGCTGGGAAAATGGTTGTTGCG-d170rev |
| TGTCTATGCGCCACAGCGATCTCAC | Primers for ChoRE-b mutation |
| CGGTATACTCCTGGCTGGGAAAATG |
| GCAGTATGAGCCTCCGGGCCAGCG | Primers for ChoRE-a mutation |
| CCTCATACACATCCCTCCCATTGGC |
| ACTCAGTGAGATCGCTGTGGCG | Primers for inverted CCAAT box mutation |
| AGTCGGGCTCCTGGTAAACAAG |
| GATGGGAGGGATGTGCACGAGGGC | Primers for CCAAT box mutation |
| AGCTGCCCGGTCCTTGTTTACCAG |
| ChIP-a, GTTCTTTCCTGCGTTATCCC | ChIP primers; refer to Fig. 9A for targeting positions of these primers. |
| ChIP-b, GACCAGGATGGGCACCAC |
| ChIP-c, AGGTTTTAGGGTCAGTGGGAT |
| ChIP-d, CTGCCCGGTCCTTGTTTAC |
| TCCAGAGCGCAACAACCAT* | ChIP primers for human Txnip promoter, use in [1] |
| AAGCAGGAGGCGGAAACGT^#^ |
| CGCACCCGAACAACAACCAT* | ChIP primer for rat Txnip promoter (*,^#^: same position) |
| AAGCGGGAGCCGGAAACGG^#^ |
| Neg-f, ATGGTTGCCACTGGGGATCT | ChIP Control primers. (NT_009759.15 on chr. 12) |
| Neg-r, TGCCAAAGCCTAGGGGAAGA |
| gccgacaggatgcagaaggagatca | β-actin  RT-PCR primers |
| AAGCATTTGCGGTGGACGATGGA |
| GGCGGGTGTCTGTCTCTGCT | Human Txnip  RT-PCR primers |
| GGCAAGGTAAGTGTGGCGGG |

[1] Ahsan MK, Masutani H, Yamaguchi Y, Kim YC, Nosaka K *et al*.(2006) [Loss of interleukin-2-dependency in HTLV-I-infected T cells on gene silencing of thioredoxin-binding protein-2.](http://www.ncbi.nlm.nih.gov/pubmed/16314839?ordinalpos=3&itool=EntrezSystem2.PEntrez.Pubmed.Pubmed_ResultsPanel.Pubmed_DefaultReportPanel.Pubmed_RVDocSum) Oncogene 25(15):2181-91.
