# Supplementary material for: Tandem ChoRE and CCAAT Motifs and Associated Factors Regulate Txnip Expression in Response to Glucose or Adenosine-Containing Molecules
Source: PLoS One. 2009 Dec 22;4(12):e8397. doi: 10.1371/journal.pone.0008397 (PMC2791861; doi:10.1371/journal.pone.0008397)
Supplement: Table S2 — Sequence information of probes used in EMSA. (0.04 MB DOC) [file pone.0008397.s009.doc]

**Table S2.** Sequence information of probes used in EMSA

| **Probes** | **Sequence** |
| --- | --- |
| **WT-a** | GACCGGGCAGCCAATGGGAGGGATGTGCACGAGGGCAGCACGAGCCTCCGGGCCAGC  TGCTGGCCCGGAGGCTCGTGCTGCCCTCGTGCACATCCCTCCCATTGGCTGCCCGGTC |
| **mCho-a** | GACCGGGCAGCCAATGGGAGGGATGTGTATGAGGGCAGTATGAGCCTCCGGGCCAGC  TGCTGGCCCGGAGGCTCATACTGCCCTCATACACATCCCTCCCATTGGCTGCCCGGTC |
| **mCAT** | GACCGGGCAGCTACTGGGAGGGATGTGCACGAGGGCAGCACGAGCCTCCGGGCCAGC  TGCTGGCCCGGAGGCTCGTGCTGCCCTCGTGCACATCCCTCCCAGTAGCTGCCCGGTC |
| **WT-b** | CAGCCAGGAGCACACCGTGTCCACGCGCCACAGCGATCTCACTGATTGGTCGGGCTC  TGAGCCCGACCAATCAGTGAGATCGCTGTGGCGCGTGGACACGGTGTGCTCCTGGCTG |
| **mCho-b** | CAGCCAGGAGTATACCGTGTCTATGCGCCACAGCGATCTCACTGATTGGTCGGGCTC  TGAGCCCGACCAATCAGTGAGATCGCTGTGGCGCATAGACACGGTATACTCCTGGCTG |
| **miCAT** | CAGCCAGGAGCACACCGTGTCCACGCGCCACAGCGATCTCACTGAGTAGTCGGGCTC  TGAGCCCGACTACTCAGTGAGATCGCTGTGGCGCGTGGACACGGTGTGCTCCTGGCTG |
| **sCho-a** | GGGAGGGATGTGCACGAGGGCAGCACGAGCCTCCGGGCC  TGGCCCGGAGGCTCGTGCTGCCCTCGTGCACATCCCTCCC |
| **msCho-a** | GGGAGGGATGTGTATGAGGGCAGTATGAGCCTCCGGGCC  TGGCCCGGAGGCTCATACTGCCCTCATACACATCCCTCCC |
| **sCho-b** | CCCAGCCAGGAGCACACCGTGTCCACGCGCCACAGCGAT  TATCGCTGTGGCGCGTGGACACGGTGTGCTCCTGGCTGGG |
| **msCho-b** | CCCAGCCAGGAGTATACCGTGTCTATGCGCCACAGCGAT  TATCGCTGTGGCGCATAGACACGGTATACTCCTGGCTGGG |
| **sNFY** | GATCTCACTGATTGGTCGGGCTC  GAGCCCGACCAATCAGTGAGATC |
